# Omega 3 fatty acids reduce myeloid progenitor cell frequency in the bone marrow of mice and promote progenitor cell differentiation

**DOI:** 10.1186/1476-511X-8-9

**Published:** 2009-03-18

**Authors:** Melinda E Varney, W Elaine Hardman, Vincent E Sollars

**Affiliations:** 1 Department of Biochemistry and Microbiology, Marshall University School of Medicine, One John Marshall Drive, Huntington, WV 25755, USA

## Abstract

**Background:**

Omega 3 fatty acids have been found to inhibit proliferation, induce apoptosis, and promote differentiation in various cell types. The processes of cell survival, expansion, and differentiation are of key importance in the regulation of hematopoiesis. We investigated the role of omega 3 fatty acids in controlling the frequency of various myeloid progenitor cells in the bone marrow of mice. Increased progenitor cell frequency and blocked differentiation are characteristics of hematopoietic disorders of the myeloid lineage, such as myeloproliferative diseases and myeloid leukemias.

**Results:**

We found that increasing the proportion of omega 3 fatty acids relative to the proportion of omega 6 fatty acids in the diet caused increased differentiation and reduced the frequency of myeloid progenitor cells in the bone marrow of mice. Furthermore, this had no adverse effect on peripheral white blood cell counts.

**Conclusion:**

Our results indicate that omega 3 fatty acids impact hematopoietic differentiation by reducing myeloid progenitor cell frequency in the bone marrow and promoting progenitor cell differentiation. Further exploration of this discovery could lead to the use of omega 3 fatty acids as a therapeutic option for patients that have various disorders of hematopoiesis.

## Background

Omega 3 and omega 6 fatty acids are essential fatty acids that must be obtained from dietary sources. Recently, the ratio of omega 3 to omega 6 fatty acids consumed in the diet has been studied in relevance to cardiac health[1], neurological health[2], and cancer prevention[1].

Omega 3 fatty acids can compete with omega 6 fatty acids for the same metabolic enzymes. Omega 3 and omega 6 fatty acids are incorporated into cell membranes either directly or after elongation and desaturation by Δ4, Δ5, and Δ6 and desaturases[3,4]. Omega 3 fatty acids have greater affinity for the Δ4 and Δ6 desaturases than omega 6 fatty acids [5-7]. Dietary linoleic acid (18 carbons, omega 6 fatty acid) is generally considered to be the major source of tissue arachidonic acid (20 carbons, omega 6 fatty acids) although meat fat can be a direct source of arachidonic acid[8]. All three major omega 3 fatty acids – α linolenic acid (18:3), eicosapentaenoic acid (20:5), and docosahexaenoic acid (22:6) – directly inhibit the production of arachidonic acid from linoleic acid[9]. Both arachidonic acid and eicosapentaenoic acid can be cleaved from the cell membrane phospholipid stores by phospholipase A2 and acted on by cyclooxygenases (either the constitutive COX1 or the inducible COX2) to produce prostaglandin precursors which are isomerized by prostaglandin synthases to produce prostaglandins. In this manner, COX converts arachidonic acid to form the 2-series prostaglandins that tend to be pro-proliferative and pro-inflammatory in most tissues[10]. Micromolar concentrations of prostaglandin E2 (PGE2) increase human myeloid progenitor cell proliferation[11]. However, COX activity on eicosapentaenoic acid forms the 3 series prostaglandins that tend to have anti-proliferative and anti-inflammatory properties[10]. In addition to prostaglandins, leukotrienes and eicosanoids are formed from fatty acids through activity of various lipoxygenases. These have been shown to have varying and sometimes controversial effects on either hematopoietic stem cell or myeloid progenitor cell differentiation[11,12]. Thus, a model system approach is needed to effectively dissect the net effect of dietary fatty acids on hematopoiesis *in vivo*.

Abnormal hematopoiesis often involves expansion of immature cells of the myeloid lineage. In these cases, progenitor cell frequency could be expanded by increased proliferation, decreased apoptosis, or both. If omega 3 fatty acids inhibit proliferation or induce apoptosis in myeloid progenitor cells, they could alleviate the cellular expansion that occurs in certain disorders of hematopoiesis. Another characteristic of these disorders is a block in differentiation. During normal hematopoiesis, a continuum of differentiation exists, such that long term hematopoietic stem cells progressively differentiate to produce terminally differentiated cells of the blood. Since omega 3 fatty acids have been shown to promote differentiation [11], they may serve as a therapeutic tool [13-16] for disorders involving the inhibition of normal differentiation.

Our results indicate that the frequency of myeloid progenitors in the bone marrow of mice fed fish oil diets is two-fold less than in those fed corn oil diets. Our data also indicate that this reduction of progenitor cells does not have an adverse effect on the number of circulating white blood cells in the periphery, even after prolonged dietary change. These results suggest a possible role for omega 3 fatty acids as therapeutic agents in disorders involving expansion of myeloid progenitors, such as the myeloid leukemias.

## Results and discussion

### High levels of omega 3 fatty acids alter the character of the myeloid progenitor cell compartment in the bone marrow

**Figure 1:**
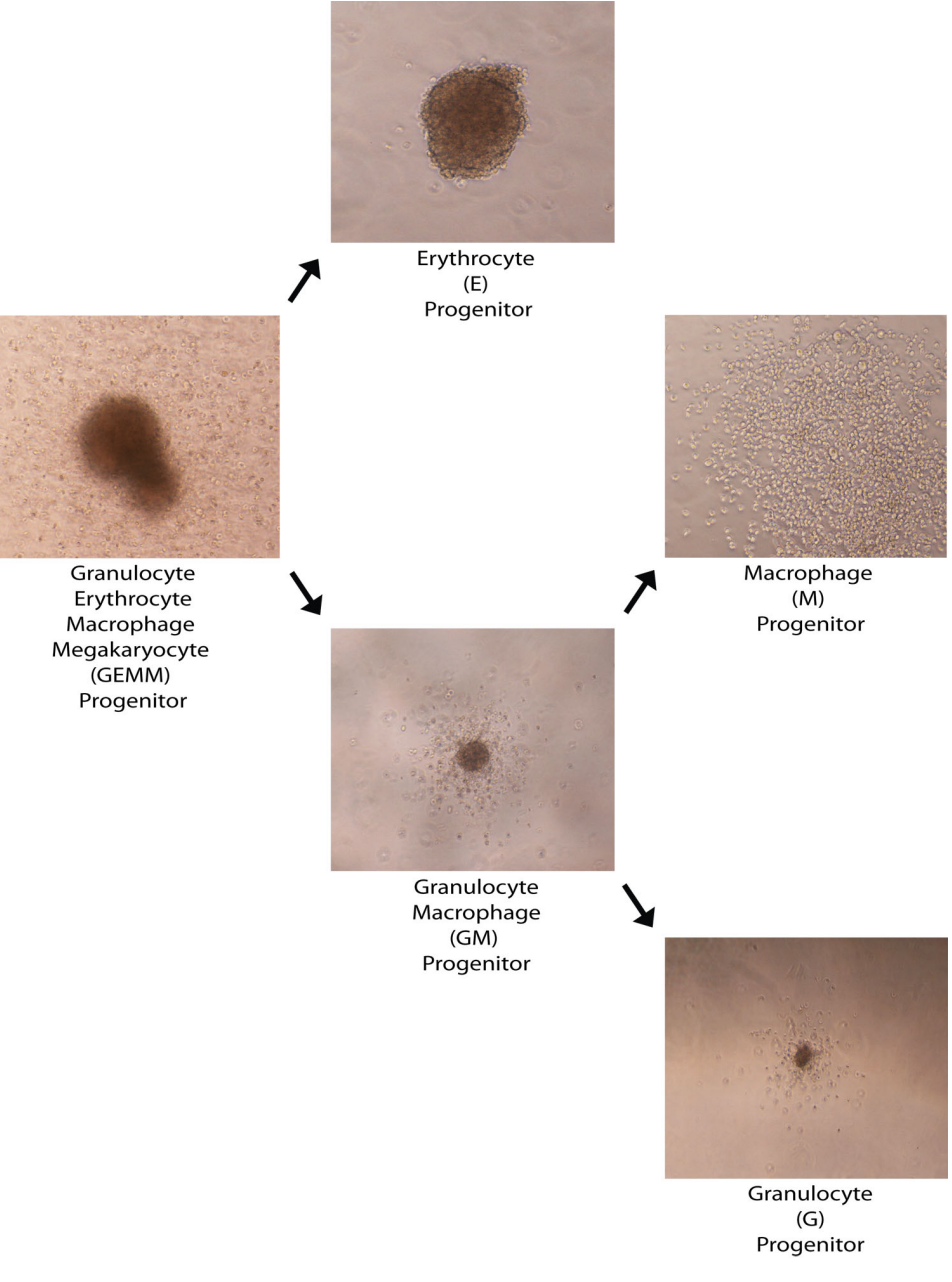
**Hematopoiesis Differentiation Continuum – Myeloid Progenitor Morphology**. Hematopoiesis exists as a continuum of differentiation. Colony morphology representing the various myeloid progenitor cells is shown (magnification = 100×) in a differentiation hierarchy beginning with the least differentiated progenitor and continuing to the most differentiated progenitor. Abbreviations: Granulocyte erythrocyte macrophage megakaryocyte (GEMM), granulocyte macrophage (GM), erythrocyte (E), granulocyte (G), macrophage (M).

**Figure 2:**
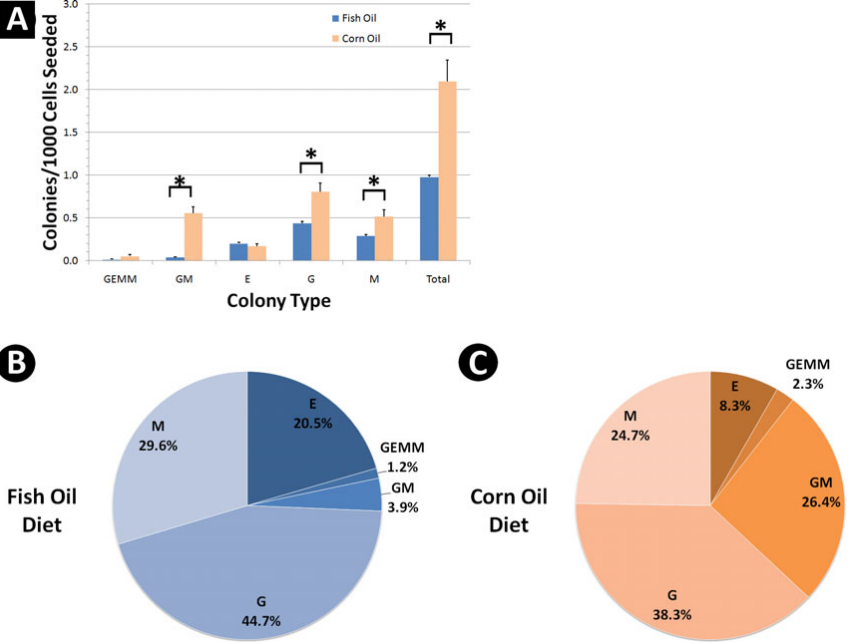
**Fish oil diets cause changes in the steady state levels of myeloid progenitors in the bone marrow**. (A) Colony forming cell (CFC) assay results of bone marrow from mice fed corn oil (high omega 6) vs. fish oil (high omega 3) diets until 60 days of age. Colonies formed represent the presence of a progenitor cell where the type of colony determines which progenitor cell type is present. Comparisons marked with (*) were found to be significantly different (p < 0.05). Error bars represent SEMs (n = 5). (B-C) Data from (A) where each colony type is shown as a proportion of total progenitor cell frequency for each diet.

Progenitors for myeloid cells in bone marrow that can be assessed by colony forming cell (CFC) assays include granulocyte erythrocyte macrophage megakaryocyte (GEMM) progenitors, granulocyte macrophage (GM) progenitors, erythrocyte (E) progenitors, granulocyte (G) progenitors, and macrophage (M) progenitors. Only these progenitors have the necessary growth factors and proliferation capacity to produce colonies in the CFC assay. Morphology of these colony types are shown in Figure 1. Mice were fed fish oil (high level omega 3 fatty acids) or corn oil (low level omega 3 fatty acids) containing diets (Tables 1 and 2). Mice that were fed the fish oil diet had a significantly lower (p = 0.010) frequency of total myeloid progenitor cells (9.78 ± 0.46 × 10^-4^) than those mice fed the corn oil diet (2.10 ± 0.55 × 10^-3^) (Fig. 2A). These results indicate that omega 3 fatty acids were inducing a reduction in total myeloid progenitors in bone marrow. GM, G, and M progenitors were significantly less (p < 0.05) frequent in the bone marrow of mice fed the fish oil diet than those fed the corn oil diet (Fig. 2A). Interestingly, E progenitors did not change in their frequency with diet, indicating omega 3 fatty acids preferentially affected levels of GM progenitors and their daughter cells (see Fig. 1). There was also a larger frequency of GEMM progenitors, which are early progenitors, in corn oil (5.16 ± 4.32 × 10^-5^) vs. fish oil (1.20 ± 1.20 × 10^-5^) fed mice. Although this was a greater than 4 fold difference in frequency, the difference was not statistically significant (p = 0.11) and the rarity of this progenitor relative to others made it difficult to accurately assay with this technique.

**Table 1:** Modified AIN-76A Diet Composition.

	**Diet composition**	
	
**Ingredient**	**% of weight**	**Amount/100 g**
Casein (protein)	20%	20 g
Sucrose	45%	45 g
Corn starch (carbs)	15%	15 g
Alphacel (fiber)	5%	5 g
Choline bitartrate	0.2%	0.2 g
DL-methionine	0.3%	0.3 g
Mineral mix	3.5%	3.5 g
Vitamin mix	1.0%	1 g
		
Fat	10%	10 g
Total	100%	100 g
		
Total fat		10 g
Total protein		20 g
Total carbohydrate		60 g

The base diet is an AIN-76A diet modified by substitution of 5% sucrose for 5% more oils to contain a total of 10% w/w oil. The mouse food recipe contains 10% fat in each diet supplied through these oils.

**Table 2:** Compositions of dietary fats (approximate %).

	**Saturated fatty acids**	**Linoleic acid (omega 6)**	**Total omega 3**	**Monounsaturated fatty acids**
Corn oil^a^	13	61	1	26
Canola oil^a^	6	20	10	62
n-3 supp.^b^	9	6	63	21

The corn oil diet is the low omega 3: omega 6 fatty acid diet containing 10% w/w corn oil as the source of all fat. The fish oil diet is our high omega 3: omega 6 fatty acids diet containing 5% w/w canola oil and 5% w/w omega 3 fatty acid supplement. The corn oil diet contains 0.1% omega 3 fatty acids and 6.1% omega 6 fatty acids, which is a relative ratio of omega 6 to omega 3 of 61: 1. The fish oil diet contains 3.65% omega 3 fatty acids and 1.3% omega 6 fatty acids, which is a relative ratio of 1: 2.8.

^a^From:

^b^From: Manufacture's certified analyses.

Analysis of the proportions of progenitor cells that make up the myeloid progenitor cell compartment in these mice revealed that mice fed a fish oil diet had a much different proportion of early and late stage progenitors compared to those fed a corn oil diet (p = 0.00015). There was a greater percentage of later stage progenitor cells (G, M, E progenitors) in fish oil diet fed mice (94.9 ± 1.6%) when compared to mice fed a corn oil diet (71.3 ± 4.8%) (Fig. 2B). Conversely, there was a greater proportion of earlier stage progenitors (GEMM and GM progenitors) in corn oil fed mice (28.7 ± 4.8%) vs. fish oil fed mice (5.15 ± 1.6%). Thus, our data indicate that the bone marrow of mice fed a high level of omega 3 fatty acids had a more differentiated myeloid progenitor cell compartment than that of mice fed a diet containing mostly omega 6 fatty acids.

### High levels of omega 3 fatty acids do not have an adverse effect on white blood cell production in the peripheral blood

**Figure 3:**
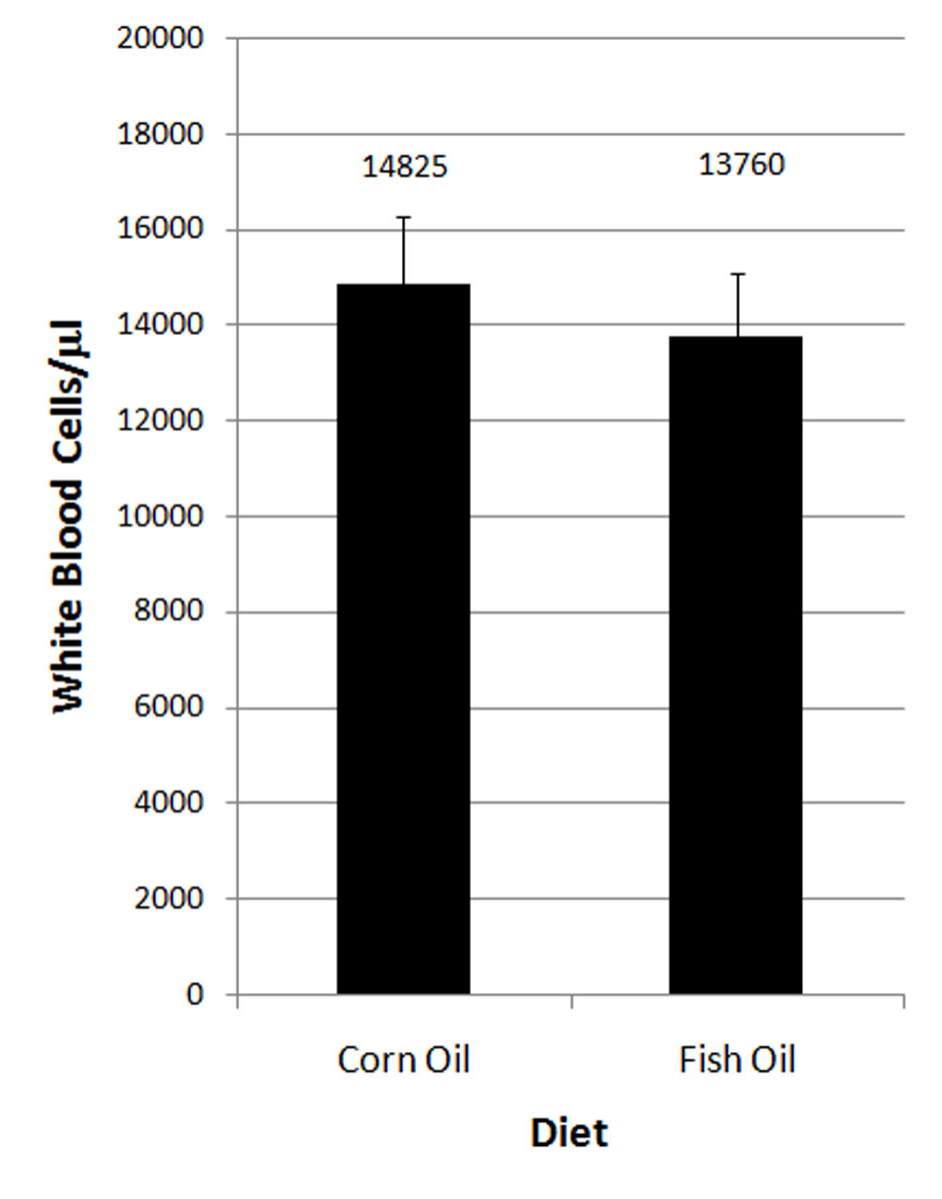
**Fish oil diets do not cause adverse reduction of white blood cell counts in the peripheral blood**. White blood cell (WBC) counts in WBC/μl from female mice on the two diets until 115 days of age (n = 4 for fish oil, n = 5 for corn oil, p = 0.602). Error bars represent SEMs.

To account for the concern that a lower progenitor cell frequency or a greater proportion of differentiated progenitor cells in the bone marrow may cause a reduction in white blood cells in peripheral blood and therefore a weakened immune system, we performed white blood cell (WBC) counts on mice fed these diets from weaning to 115 days. Results indicated no significant differences in peripheral WBC counts in corn oil fed mice (14,825 WBC/μl) and fish oil fed mice (13,760 WBC/μl) (Fig. 3). Thus, fish oil diets induce a homeostatic condition of decreased progenitor cell frequency in the myeloid compartment of the bone marrow without adverse effects on WBC production.

## Conclusion

Our results indicate that omega 3 fatty acids reduce total myeloid progenitor cell frequency and promote differentiation of specific progenitor cell types in the bone marrow of mice. Furthermore, this reduction of progenitors does not have an adverse effect on levels of circulating white blood cells. Our data indicate that omega 3 fatty acid effects are particular to granulocyte and macrophage progenitor cells rather than erythrocyte maturation. The effects of omega 3 fatty acids may occur as early in maturation as the GEMM progenitor stage, and then be propagated to the GM and later progenitor stages. While our results indicate a difference at the GM progenitor stage, they are also suggestive, although inconclusive, of a difference at the GEMM stage. We are currently examining this question with ongoing experiments to look at the earliest stem and progenitor cell stages of hematopoiesis.

Examination of the effects of omega 3 fatty acids on hematopoiesis could lead to potential therapeutic options for patients with disorders of the blood. Future work would include investigating the mechanisms of action for these events. Mechanisms by which omega 3 fatty acids have been shown to inhibit proliferation, induce apoptosis, and promote differentiation in many cancers include the regulation of signaling pathways and gene expression by peroxisome proliferator receptor activator γ(PPARγ), for which omega 3 fatty acids are natural ligands[17]. Another mechanism of action of omega 3 fatty acids includes inhibition of cyclooxygenase 2 (COX2), which is upregulated in various cancers and is known to have proproliferative and antiapoptotic effects [18-26]. Furthermore, the combination of activating PPARγ and inhibition of COX2 expression has recently been shown to inhibit proliferation and induce apoptosis in pancreatic cancer[27]. We plan to explore these, among other mechanisms, using our animal model.

Regardless of the mechanisms by which omega 3 fatty acids reduce and differentiate myeloid progenitor cells in the bone marrow of mice, these dietary agents serve as a promising option for therapy. The inhibitory effect of omega 3 fatty acids on immune system function[11,28] has led to the use of fish oils that are high in these fatty acids in the management of several inflammatory and autoimmune diseases[28]. Suppression of omega 6 derived eicosanoids has been proposed as a strategy for chemoprevention and as an adjunct for treatment of cancer [13-16]. Our results suggest that using omega 3 fatty acids to more rapidly differentiate myeloid progenitor cells may slow progression of disorders of hematopoiesis, including leukemias, and restore a more normal myeloid progenitor cell compartment in the bone marrow.

## Methods

### Animals

Mice were housed in the AAALAC accredited animal facilities of the Marshall Universitiy School of Medicine. All animal use and care was approved by the Marshall University Institutional Animal and Use Committee. The mice were housed 3 to 4 in a cage and individually numbered for identification.

### Diet

The base diet was an AIN-76A diet modified by substitution of 5% sucrose for 5% more oils to contain a total of 10% w/w oil (Tables 1 and 2). The fish oil diet contained 3.65% n-3 fatty acids and 1.3% n-6 fatty acids. The corn oil diet contained 0.1% n-3 fatty acids and 6.1% n-6 fatty acids. Diets were prepared in the Marshall University School of Medicine animal diet prep room. Diet composition is shown in Table 1 and was formulated to be isocaloric, isonutrient and relevant to human consumption. The AIN-76A diet is adequate for the nutritional support of the mice[29]. The dry ingredients of the diet were obtained in bulk from MP Biomedicals (Solon, Ohio), sugar, corn and canola oil were purchased locally (100% canola oil, 100% corn oil, no additives or preservatives). The omega 3 supplement (OmegaRx Liquid) was purchased from Zone Labs, Danvers, MA. Batches of diet were prepared as needed, about every two weeks. The diet mixture was pressed into trays and cut into small squares. Individual cage sized portions (25–30 g) were stored in sealed containers at -20°C to prevent oxidation of the fat and bacterial growth in the food. Mice had free access to food and water and were fed fresh food 5 days per week. Food removed from cages was discarded.

### Colony Forming Cell (CFC) Assays

FVB X sv129 F_1_hybrid mice were fed either a fish oil diet (n = 5) or a corn oil diet (n = 5) from weaning until 60 days old. CFC assays were then performed upon bone marrow isolated from these mice as previously discussed[30,31]. Briefly, bone marrow was harvested by flushing from the femurs with Iscove's Modified Dulbecco's Medium (IMDM) and cells were counted and seeded in 4 well plates in at a density determined empirically by a pilot study conducted with a broad and consistent range of seeding densities. After linear response of colony production to seeding density was ensured by the pilot study, seeding densities of 2.09 × 10^4 ^(fish oil) and 7.75 × 10^3 ^(corn oil) cells/well were chosen to produce 10–30 colonies for each well. Bone marrow was cultured in 1% semi-solid Methocult M3434 (StemCell Technologies), supplemented with 0.4% autochthonous sera. Cells were incubated for 6–7 days to allow colony formation. Colonies with a minimum cell number of 20 were scored as positive using an inverted microscope at 40× magnification. Colonies were counted based on morphological features that are associated with each progenitor type. Student's *t *type tests were used to detect differences between the experimental groups (n = 5).

### White Blood Cell Counts

White blood cell counts were performed on EDTA anticoagulated peripheral blood from tail veins of corn and fish oil fed female mice aged 115 days. Fish and corn oil diets were instituted after weaning. Student's *t *type tests were used to detect differences between the experimental groups.

## Competing interests

The authors declare that they have no competing interests.

## Authors' contributions

MEV was responsible for the large part of data acquisition. MEV and VES analyzed data and wrote this manuscript. WEH provided the mice with appropriate diets and intellectual contributions on study design. VES and MEV provided intellectual contribution on study design as well. VES coordinated the project.
